# Genetic evidence of regional circulation of Crimean-Congo hemorrhagic fever virus in ixodid ticks from southern Kazakhstan

**DOI:** 10.3389/fvets.2025.1623822

**Published:** 2025-10-03

**Authors:** Maxat Berdikulov, Kydyrbay Maikhin, Talgat Karibayev, Kanat Kalkabayev, Botagoz Kazybay, Raikhan Nissanova, Abzal Makhmutov, Nurkuisa Rametov, Abishov Abdikalyk, Sarsenbay Abdrakhmanov, Dong-Kun Yang, Gulzhan Mussayeva

**Affiliations:** ^1^National Veterinary Reference Center, Almaty, Kazakhstan; ^2^Kazakh Scientific Research Veterinary Institute LLP, Almaty, Kazakhstan; ^3^Kazakh National Agrarian Research University, Faculty of Veterinary Science and Zooengineering, Almaty, Kazakhstan; ^4^Tecton Analytics LLP, Astana, Kazakhstan; ^5^Diavak-ABN Scientific and Production Center, Almaty, Kazakhstan; ^6^S. Seifullin Kazakh Agrotechnical University, Animal science and veterinary institute, Astana, Kazakhstan; ^7^Animal and Plant Quarantine Agency, Gimcheon, Republic of Korea

**Keywords:** arbovirus, lumpy skin disease, vector, virus, tick, Crimean-Congo hemorrhagic fever, Schmallenberg virus, bluetongue virus

## Abstract

**Introduction:**

Arthropod-borne viruses (arboviruses) pose a growing threat to livestock and human health across Central Asia. This study aimed to assess the presence and genetic diversity of arboviral pathogens—Crimean-Congo hemorrhagic fever virus (CCHFV), Bluetongue virus (BTV), Schmallenberg virus (SBV), and lumpy skin disease virus (LSDV)—in ixodid ticks livestock in southern Kazakhstan.

**Methods:**

A total of 3,281 adult ticks were collected from three regions (Turkestan, Zhambyl, and Kyzylorda) and identified morphologically. Molecular screening was performed using real-time and nested RT-PCR.

**Results:**

CCHFV RNA was detected exclusively in female ticks from the Turkestan region, with Dermacentor pictus showing the highest infection rate (21.05%), followed by Hyalomma anatolicum, Dermacentor marginatus, and Hyalomma scupense. No viral RNA was detected for BTV, SBV, or LSDV.

**Discussion:**

Phylogenetic analysis based on partial S and L segments revealed that the Kazakhstani isolates clustered within Asia-1 and Asia-2 genotypes and shared high sequence identity with regional strains from Uzbekistan, Turkmenistan, and China, supporting transboundary virus circulation. These findings provide additional molecular evidence of localized CCHFV activity in livestock-associated ticks in southern Kazakhstan, expanding current knowledge on the virus’s geographic distribution and genetic diversity.

## 1 Introduction

Economic growth in the livestock sector under modern market conditions largely depends on the effectiveness of measures aimed at reducing animal losses caused by non-contagious, infectious, and viral diseases (1). In this context, viral infections play a crucial role, as they not only inflict damage on livestock industry also pose a threat to public health. Among the infectious diseases affecting animals, arboviruses cause substantial economic damage (2). The most significant arboviral threats in Kazakhstan include bluetongue virus (BTV), lumpy skin disease virus (LSDV), Schmallenberg virus (SBV), and Crimean-Congo hemorrhagic fever virus (CCHFV) (3–5).

Arboviruses transmitted by arthropods pose a global public health challenge (6, 7). In recent years, there has been an increase in the incidence of arboviral infections, an expansion of their geographic distribution, and a growing burden on healthcare systems (8, 9). These viruses exhibit similar epidemiological characteristics, transmission routes, and clinical manifestations, although the severity of complications may vary (10, 11). Clinical signs of arboviral infections are often non-specific, mild, or even absent; however, in some cases, they can lead to severe complications (12, 13).

Arboviruses are maintained in natural transmission cycles between vertebrate hosts and hematophagous arthropods, such as mosquitoes, midges, gnats, and ticks (14). For the transmission cycle to be completed, arboviruses must reach a sufficiently high level of viremia in the vertebrate host to effectively infect the arthropod during blood feeding (15, 16).

Investigation of ixodid ticks for the carriage of dangerous viral pathogens—such as BTV, CCHFV, LSDV, and CCHFV—represents a critical task from both scientific and practical perspectives (17, 18). Over 135 arboviruses capable of causing human diseases have been identified (19). These infections can occur asymptomatically or lead to fulminant, fatal outcomes, with clinical manifestations ranging from systemic febrile syndromes to hemorrhagic fevers (20, 21).

CCHFV remains one of the most pressing issues in Kazakhstan (22). The CCHFV causes outbreaks of hemorrhagic fever, multiple studies report case fatality rates ranging from 25 to 60% (23, 24). Genetically, the CCHFV belongs to the Nairovirus family and is characterized by a high degree of genetic diversity. Its genome consists of three segments of single-stranded RNA: the small (S), medium (M), and large (L) segments (25). The disease is reported in Africa, Asia, and Europe, where the mortality rate reaches up to 30% (23, 26).

Based on the analysis of the nucleotide sequences of the S and L segments of the CCHFV genome, seven genotypes have been identified: Africa-1, Africa-2, Africa-3, Asia-1, Asia-2, Euro-1, and Euro-2 (27). CCHFV has been reported in Kazakhstan since 1948, with active natural foci identified in the Zhambyl, Turkestan, and Kyzylorda regions (28). The epidemiological situation in these regions remains unstable, with approximately 16 clinical cases reported annually and an average case fatality rate of 14.8% (29).

Genetic analysis of the CCHFV isolated from the southern regions of Kazakhstan has revealed its affiliation with the Asia-1 and Asia-2 genotypes, indicating genetic relatedness to strains from Uzbekistan, Tajikistan, and China (30).

LSDV was first reported in Kazakhstan in 2016 in the Atyrau region (4). Given the high density of livestock and the presence of vectors, further spread of this infection could result in significant socioeconomic consequences (31).

Studies on the causative agent of bluetongue remain preliminary, and the virus associated with Schmallenberg disease has not yet been isolated. Moreover, no systematic monitoring of the epizootic situation for these diseases has been conducted in Kazakhstan, thereby limiting our understanding of their impact in the region.

The aim of this study was to investigate the distribution of four high-priority arboviral infections—CCHFV, BTV, SBV, and LSDV—relevant to both public and animal health, in the southern regions of Kazakhstan, as well as to identify their vectors among ixodid ticks collected from livestock.

The study of arboviral infections and their vectors is crucial for developing effective control and prevention strategies against diseases that threaten both animal husbandry and public health. The data obtained can be used to enhance epidemiological surveillance systems and mitigate the risks of arbovirus transmission in the region. However, the absence of molecular surveillance data on arboviral pathogens in ticks from Kazakhstan continues to limit regional risk assessment and preparedness. This study addresses this gap through genetic detection and characterization of circulating arboviruses in livestock-associated ixodid ticks.

## 2 Materials and methods

### 2.1 Sample collection and preparation

Sample collection and preparation were conducted in accordance with the WOAH Terrestrial Manual 2024 (Chapter 1.1: Specimen Collection, Submission, and Preparation). Biological specimens of ixodid ticks were collected from livestock such as cattle, sheep, and goats across farms and private holdings in three regions of Kazakhstan: Zhambyl (382 samples, 11.7%), Turkestan (2,690 samples, 81.9%), and Kyzylorda (209 samples, 6.4%) in 2024. In the Turkestan region, tick sampling was additionally stratified by administrative district, with collections performed in Shardara, Arys, Maktaaral, and Baitibek districts to enable localized analysis of virus circulation patterns. Sampling was conducted as single-timepoint examinations, involving 10 to 30 animals per herd. Particular attention was given to body regions where ticks are known to commonly aggregate, including the neck, axillae, groin, perianal area, udder, and tail base.

Host preference analysis was performed by examining 10% of animals within selected herds. The average tick burden per host species was calculated using the formula:


[B=\frac{K\times100}{S},]

where BB = host species, KK = number of ticks on the host species, and SS = total ticks collected from all host species.

Tick collection was conducted exclusively from cattle in private household farms located in endemic southern regions of Kazakhstan. A total of 3,281 adult ticks were collected, including 382 samples (11.7%) from Zhambyl, 2,690 samples (81.9%) from Turkestan, and 209 samples (6.4%) from Kyzylorda regions. Following morphological identification, the collected specimens were classified into nine tick species belonging to the family Ixodidae.

Ticks were manually removed using surgical forceps to avoid damaging mouthparts and preserved in 70% ethanol. Morphological identification was confirmed microscopically using taxonomic keys (32). All procedures were performed in accordance with biosafety protocols 3.1.1027-01: Guidelines for the Collection and Laboratory Analysis of Arthropod Vectors (33).

### 2.2 Nucleic acid extraction

A total of 3,281 adult ticks were analyzed, including *Hyalomma scupense* (27.5%), *Hyalomma anatolicum* (19.6%), *Dermacentor marginatus* (15.2%), *Boophilus calcaratus* (6.6%), *Dermacentor pictus* (5.6%), *Haemaphysalis otophila* (0.7%), *Haemaphysalis punctata* (0.5%), *Rhipicephalus bursa* (0.4%), *Dermacentor niveus* (0.2%), *Argas persicus* (23.7%), and *Alveonasus lahorensis* (0.03%). Ticks were homogenized in 2.0 mL DNase/RNase-free tubes (Eppendorf) with zirconia-silica beads (6 mm) and DMEM medium (Capricorn Scientific) using a LabSafer TS-48/64 homogenizer. Homogenates were freeze-thawed three times, clarified by centrifugation (600 × *g*, 10 min), and supernatants pooled by species for downstream analysis.

Total nucleic acids were extracted using the PureLink Microbiome DNA/RNA Purification Kit (Invitrogen, USA) following the manufacturer’s protocol.

### 2.3 Viral amplification and detection

Detection of arboviruses in tick samples was performed using different PCR-based approaches depending on the viral genome type. Ticks were pooled into groups of 3–5 individuals according to species, sex, and sampling location prior to nucleic acid extraction and subsequent molecular analysis. For the RNA viruses—CCHFV, SBV, and BTV—we employed either one-step RT-PCR or nested RT-PCR using virus-specific primers to enhance analytical sensitivity and detect low viral loads. Real-time RT-PCR (AmpliSens® kits, Russia) was additionally used for confirmation of CCHFV-positive samples. For the DNA virus LSDV, conventional PCR targeting the P32 gene was performed using primers described by El-Nahas et al. (34). For phylogenetic analysis, partial fragments of the S segment (~536 bp) and L segment (~476 bp) of the CCHFV genome were amplified. All procedures were performed in a BSL-3 facility compliant with WHO biosafety guidelines.

BTV and SBV were detected by one-step reverse transcription quantitative PCR (RT-qPCR), whereas LSDV, a DNA virus was tested by a quantitative PCR assay. We used commercial kits specific for each virus: Virotype® BTV RT-PCR Kit and Virotype® SBV RT-PCR Kit (Qiagen, Germany); and VetMAX® LSDV/Capripox PCR Kit (Thermo Fisher Scientific, USA). Reactions were performed according to the manufacturer’s instructions.

### 2.4 Nested RT-PCR for CCHFV

Viral RNA was amplified using the BioMaster One-Step RT-PCR–Extra kit (2×) (Biolabmix, Russia) following the manufacturer’s instructions. The first-round RT-PCR was carried out in a 20 μL reaction volume containing 5 μL of extracted RNA and the following primer sets targeting the S, M, and L segments of the CCHFV genome (Table 1). Thermal cycling conditions were as follows: reverse transcription at 50 °C for 30 min; initial denaturation at 95 °C for 5 min; followed by 35 cycles of 95 °C for 40 s, 58 °C for 40 s, and 72 °C for 90 s; with a final extension at 72 °C for 5 min. The second-round PCR was performed in a 20 μL reaction containing 5 μL of the first-round product and the following in Table 1 primers. Thermal cycling conditions were: initial denaturation at 95 °C for 5 min; followed by 35 cycles of 95 °C for 1 min, 49 °C for 40 s, and 72 °C for 90 s; with a final elongation step at 72 °C for 5 min.

**Table 1:** Primer sequences and nested RT-PCR conditions.

Primer	Nucleotides sequence (5′–3′)	Target	Amplicon size (bp)	Reference
Primers for the first round of amplification				
S-rna-CCHF-F1	acgcccacagtgttctcttgagtg	S segment	738	Nurmakhanov et al. (42)
S-rna-CCHF-R1	caaggcctgttgcracaagtgctat			
M-CCHF-Kuhn-F	caaagaaatacttgcggcacg	M segment	956	Kuhn et al. (45)
M-CCHF-NCB-R1	cctyttacaccaytctagyargccttc			
L-CCHF-NCB-F1	cttamgaggatgctrtctgacaa	L segment	821	Nurmakhanov et al. (42)
L-CCHF-NCB-R1	ttgttagarccrtataagaatgttga			
Primers for the second round of amplification				
Burt-CCHF-F1	tggacaccttcacaaactc	S segment	536	Burt et al. (46)
Burt-CCHF-R1	gacaaattccctgcacca			
M-CCHF-NCB-F2	tcagtacgtaagtgttaactttgag	M segment	847	Nurmakhanov et al. (42)
M-CCHF-NCB-R2	ccttgaggnaangtcaagattat			
L-CCHF-NCB-F2	tggagayggtgatgtgtttacagc	L segment	608	
L-CCHF-NCB-R2	gctgcatatgyctttctatycctgt	S segment		

PCR products were analyzed by electrophoresis on a 1.5% agarose gel stained with ethidium bromide. Visualization was performed using the GelDoc imaging system (Bio-Rad, USA) and analyzed with Image Lab™ Software (Bio-Rad, USA).

### 2.5 Sequencing and phylogenetic analysis

The sequences were assembled using SeqMan software (DNASTAR, USA; version 6.1) and aligned using the MAFFT algorithm. The reference sequences published in the manual by Lukashev et al. (47) were used for the analysis. Evolutionary models were estimated in the MEGA 11 program, the best-fitting model for nucleotide sequences was Tamura et al. (35). The Tamura–Nei model was selected as the best-fit nucleotide substitution model based on Bayesian Information Criterion (BIC). Phylogenetic trees were constructed using the maximum likelihood method, incorporating the nearest neighbor interchange (NNI) heuristic algorithm and an automatically generated initial tree based on the default NJ/MP approach. Branch reliability was measured using bootstrap analysis with 1,000 repetitions. The percentage identity between sequences was calculated in the MegAlign program, which is part of the Lasergene 6.0 package (DNASTAR, USA). Phylogenetic trees based on the partial S and L genome segments of the studied CCHFV isolates are presented in Supplementary Figures 1, 2. The primer sequences used for amplification of the target regions of viral genomes, as well as the PCR conditions, are presented in Table 1.

## 3 Results

### 3.1 Tick species composition and regional distribution

To assess the prevalence of arboviral infections in ixodid ticks, a comprehensive molecular screening was conducted using real-time reverse transcription PCR (RT-qPCR) for the detection of viral RNA/DNA, including BTV, SBV, LSDV, and CCHFV. All samples tested negative for BTV, SBV, and LSDV. Only CCHFV RNA was detected in the tick samples, and therefore, only this virus was further analyzed by nested RT-PCR and sequencing.

**Figure 1:**
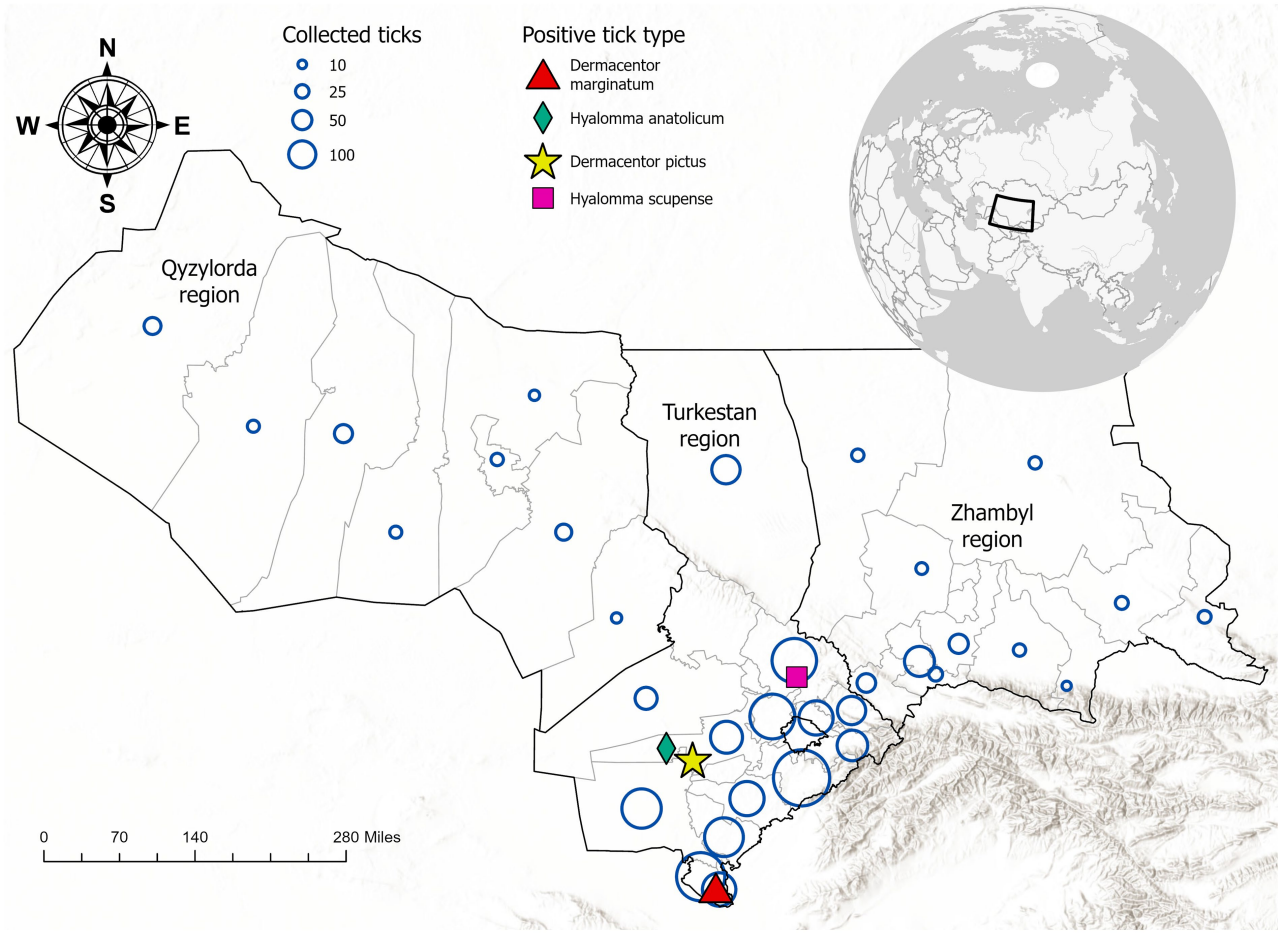
Geographic distribution of ixodid tick collection sites and detection of Crimean-Congo hemorrhagic fever virus (CCHFV)-positive tick species in southern Kazakhstan. Blue circles represent the number of ticks collected per site, with larger circles indicating higher sampling density. Colored symbols indicate tick species that tested positive for CCHFV RNA: red triangle (*Dermacentor marginatum*), green diamond (*Hyalomma anatolicum*), pink square (*Dermacentor pictus*), and yellow star (*Hyalomma scupense*). The inset map shows the location of Kazakhstan in Central Asia.

A total of 3,281 adult ticks (2,627 females and 654 males) were collected from cattle herds across three regions of Kazakhstan: Zhambyl (382 samples, 11.7%), Turkestan (2,690 samples, 81.9%), and Kyzylorda (209 samples, 6.4%). Ticks were sampled from livestock using standardized protocols, focusing on body regions with high tick aggregation (e.g., neck, axillae, groin, udder, and tail base). All specimens were transported to the National Reference Center for Veterinary Medicine for further analysis. The sampling location and quantity are shown in Figure 1.

Morphological identification confirmed the presence of nine species from the Ixodidae family—*Dermacentor marginatus* (*D. marginatus*)*, Dermacentor pictus* (*D. pictus*)*, Dermacentor niveus* (*D. niveus*)*, Hyalomma anatolicum* (*H. anatolicum*), *Haemaphysalis punctata* (*Hae. punctata*)*, Hyalomma scupense* (*H. scupense*), *Rhipicephalus bursa* (*R. bursa*), *Boophilus calcaratus* (*B. calcaratus*), and *Haemaphysalis otophila* (*Hae. otophila*). *Additionally,* two species from the *Argasidae* family were identified: *Alveonasus lahorensis* (*A. lahorensis*) and *Argas persicus* (*A. persicus*).

Epidemiological surveys and sample collection were conducted during scientific expeditions to Zhambyl, Turkestan, and Kyzylorda regions. The collected ticks were screened for arboviral pathogens using RT-qPCR assays targeting conserved genomic regions of BTV, SBV, LSDV, and CCHFV.

The species distribution of ticks in southern Kazakhstan revealed the following prevalence *- H. scupense* (27.5%), *H. anatolicum* (19.6%), *D. marginatus* (15.2%), *B. calcaratus* (6.6%), *D. pictus* (5.6%), *Hae. Otophila* (0.7%), *H. punctata* (0.5%), *R. bursa* (0.4%), and *D. niveus* (0.2%). Among *Argasidae, Argas persicus* was the most abundant (23.7%), while *A. lahorensis* was rare (0.03%).

These findings highlight the dominance of *Hyalomma* and *Dermacentor* species as potential vectors for arboviral transmission in the region. The high prevalence of *H. scupense* and *H. anatolicum* is particularly noteworthy, given their known role in transmitting CCHFV and other zoonotic pathogens.

To assess the prevalence and distribution of arboviral infections in Kazakhstan, tick pools were collected from three regions—Turkestan, Kyzylorda, and Jambyl—during 2024. The collected ticks were grouped by species and sex, and each pool was subjected to real-time RT-PCR (RT-qPCR) for the detection of BTV, SBV, and LSDV, and nested RT-PCR for the detection of CCHFV, including BTV, SBV, LSDV, and CCHV. Table 2 presents a detailed breakdown of the total number of ticks per species and region, along with the corresponding real-time RT-PCR and nested RT-PCR results. While real-time RT-PCR results for BTV, SBV, and LSDV were predominantly negative, CCHFV positivity varied across species and regions, with notable differences observed between female and male tick pools. These findings underscore the regional and taxonomic heterogeneity of arboviral infections in ticks and emphasize the need for ongoing surveillance in these endemic areas.

**Table 2:** Tick taxonomy and CCHFV positivity.

Family	Genus	Species	No. of ticks—female	No. of ticks—male	No. of positive pools	CCHFV positive (%)
Ixodidae	*Dermacentor*	*D. pictus*	128	56	21/113	18.58%
Ixodidae	*Dermacentor*	*D. niveus*	4	1	–	–
Ixodidae	*Dermacentor*	*D. arginatus*	397	102	14/279	5.02%
Ixodidae	*Rhipicephalus*	*B. alcaratus*	132	83	–	–
Ixodidae	*Rhipicephalus*	*R. bursa*	11	0	–	–
Ixodidae	*Haemaphysalis*	*H. otophila*	2	0	–	–
Ixodidae	*Haemaphysalis*	*H. punctata*	17	0	–	–
Ixodidae	*Hyalomma*	*H. scupense*	751	150	4/88	4.55%
Ixodidae	*Hyalomma*	*H.anatolicum*	598	48	15/435	3.45%
Argasidae	*Alveonasus*	*A. lahorensis*	1	0	–	–
Argasidae	*Argas*	*A. persicus*	566	211	–	–

Percent positivity is based on the number of CCHFV-positive pools out of the total pools tested. Individual ticks were not tested separately.

### 3.2 Detection of pathogens by PCR

Tick pools (grouped by species and sex) for the presence of viruses from Turkestan, Kyzylorda, and Zhambyl regions were screened by RT-PCR for four arboviruses: CCHFV, BTV, SBV, and LSDV (Table 3). CCHFV was the only virus detected among all samples; no pools tested positive for BTV, SBV, or LSDV. Notably, all CCHFV-positive pools were from the Turkestan region and consisted of female ticks, with *Dermacentor pictus* showing the highest infection rate among the species tested.

**Table 3:** Tick species distribution and arbovirus RT-PCR results in three regions of Kazakhstan.

Region	Sex	Virus	Total ticks	*A. lahorensis*	*D. pictus*	*D. niveus*	*H. scupense*	*H. anatolicum*	*B. calcaratus*	*D. marginatus*	*R. bursa*	*A. persicus*	*Hae. otophila*	*Hae. punctata*
Turkestan	Female		2,137	1	113	–	582	435	119	279	5	566	20	–
		BTV	–	–	–	–	–	–	–	–	–	–	–	–
		SBV	–	–	–	–	–	–	–	–	–	–	–	–
		LSDV	–	–	–	–	–	–	–	–	–	–	–	–
		CCHFV	–	–	21/113 (18.6%)	–	4/88 (4.6%)	15/435 (3.5%)	–	14/279 (5.0%)	–	–	–	–
	Male		548	–	54	–	114	20	50	99	–	211	–	17
		BTV	–	–	–	–	–	–	–	–	–	–	–	–
		SBV	–	–	–	–	–	–	–	–	–	–	–	–
		LSDV	–	–	–	–	–	–	–	–	–	–	–	–
		CCHFV	–	–	–	–	–	–	–	–	–	–	–	–
	Total		2,685	1	167	–	696	455	169	378	5	777	20	17
Kyzylorda	Female		151	–	13	4	9	111	3	11	–	–	–	–
		BTV	–	–	–	–	–	–	–	–	–	–	–	–
		SBV	–	–	–	–	–	–	–	–	–	–	–	–
		LSDV	–	–	–	–	–	–	–	–	–	–	–	–
		CCHFV	–	–	–	–	–	–	–	–	–	–	–	–
	Male		58	–	2	1	–	22	33	–	–	–	–	–
		BTV	–	–	–	–	–	–	–	–	–	–	–	–
		SBV	–	–	–	–	–	–	–	–	–	–	–	–
		LSDV	–	–	–	–	–	–	–	–	–	–	–	–
		CCHFV	–	–	–	–	–	–	–	–	–	–	–	–
	Total		209	–	15	5	9	133	36	11	–	–	–	–
Zhambyl	Female		339	2	160	52	10	107	6	–	–	2	–	–
		BTV	–	–	–	–	–	–	–	–	–	–	–	–
		SBV	–	–	–	–	–	–	–	–	–	–	–	–
		LSDV	–	–	–	–	–	–	–	–	–	–	–	–
		CCHFV	–	–	–	–	–	–	–	–	–	–	–	–
	Male		43	–	36	4	–	3	–	–	–	–	–	–
		BTV	–	–	–	–	–	–	–	–	–	–	–	–
		SBV	–	–	–	–	–	–	–	–	–	–	–	–
		LSDV	–	–	–	–	–	–	–	–	–	–	–	–
		CCHFV	–	–	–	–	–	–	–	–	–	–	–	–
	Total		382	2	196	56	10	110	6	–	–	2	–	–

RT-PCR positivity is expressed as the number of positive pools out of the total number of pools tested for each tick species. All tick samples were tested in pools (3–5 individuals per pool); individual ticks were not tested separately.

The results presented in Table 3 indicate a marked heterogeneity in both tick abundance and arboviral infection rates across the three regions examined. In the Turkestan region—the area with the highest tick yield (2,685 individuals)—female pools exhibited notable rates of CCHFV positivity. CCHFV RNA was most frequently detected in *D. pictus,* with progressively lower positivity rates observed in *H. anatolicum, H. scupense,* and *D. marginatus*.

In both the Kyzylorda and Jambyl regions, overall tick numbers and the detection of arboviral infections were considerably lower than in Turkestan. This disparity suggests that Turkestan may represent a potential hotspot for CCHFV transmission, particularly within female tick populations of specific species. The absence of evidence for other arboviruses further underscores the predominant epidemiological relevance of CCHFV in these settings. Of the four targeted arboviruses, only CCHFV RNA was detected in the analyzed tick pools. All samples tested negative for BTV, SBV, and LSDV by PCR. Therefore, sequencing and phylogenetic analysis were conducted exclusively for CCHFV-positive samples.

### 3.3 Taxonomic and sex-specific distribution of CCHFV positivity

Detailed analysis of CCHFV positivity by taxonomic classification and sex revealed notable differences among tick species. Within the Ixodidae family, the highest infection rate was observed in female *D. pictus* (18.58%), followed by *D. marginatus* (5.02%), *H. anatolicum* (3.45%), and *H. scupense* (4.55%). No CCHFV RNA was detected in male ticks or in any specimens from the Argasidae family. These findings are summarized in Table 2, which presents species-level tick abundance and CCHFV positivity rates based on pooled molecular screening.

The results presented in Table 2 highlight marked interspecific variation in CCHFV positivity among female Ixodidae ticks. Notably, *D. pictus* and *D. marginatus* exhibited higher rates of infection compared to other taxa, suggesting a potential role as primary vectors in the region. In contrast, ticks from the Argasidae family showed no detectable CCHFV RNA, indicating their limited involvement in the virus’s local transmission cycle. These data underscore the importance of species- and sex-specific surveillance in understanding the ecology of tick-borne viruses.

### 3.4 Site-specific CCHFV detection in Turkestan region

To further examine localized patterns of CCHFV circulation, PCR assays were conducted on ticks collected from four districts within the Turkestan region. The highest CCHFV positivity was observed in *D. pictus* from Shardara District (20.5%), followed by *H. anatolicum* from Arys District (15.0%), *D. marginatus* from Maktaaral District (14.3%), and *H. scupense* from Baitibek District (16.7%) (Table 4).

**Table 4:** PCR diagnosis of arboviral infections in ticks collected from Turkestan region.

No.	Sampling location	No. of ticks collected	Tick species	Total pools tested	No. of PCR-positive pools^a^	Positivity rate (%)
1	Turkestan region, Maktaaral district, Kazibek village	151	*Dermacentor marginatus*	21	3	14.3%
2	Turkestan region, Shardara district, Dostyk village	209	*Dermacentor pictus*	44	9	20.5%
3	Turkestan region, Arys district, Baiyr village	137	*Hyalomma anatolicum*	20	3	15.0%
4	Turkestan region, Baitibek district, Tegisshil village	263	*Hyalomma scupense*	12	2	16.7%

a Positivity is calculated as the number of positive pools divided by the total number of pools tested for each species. Individual ticks were not tested separately.

The results presented in Table 4 demonstrate considerable variation in CCHFV positivity among tick species across different localities within the Turkestan region. Notably, *D. pictus* showed the highest infection rate (20.5%), suggesting a prominent role in virus maintenance. *D. marginatus*, *H. anatolicum*, and *H. scupense* also exhibited moderate positivity rates (14.3–16.7%), highlighting species-specific and ecological contributions to virus circulation.

A total of 97 out of 760 ticks (12.7%) tested positive for CCHFV RNA based on pooled analysis. These findings underscore the importance of tick species composition, local environmental factors, and host–vector interactions in shaping arbovirus transmission dynamics. The data support targeted surveillance and vector control strategies in high-risk districts of southern Kazakhstan.

### 3.5 Sequencing and phylogenetic analysis

To confirm the identity of the detected viral RNA and assess the genetic diversity of circulating CCHFV strains, representative PCR-positive samples (*n* = 97) were subjected to Sanger sequencing. Phylogenetic trees were constructed in MEGA v12 using the maximum likelihood method with the Tamura-Nei substitution model, identified as the best-fit model based on Bayesian Information Criterion (BIC) scores. Bootstrap analysis with 1,000 replicates was performed to evaluate the robustness of the tree topology. Reference sequences were retrieved from GenBank and included strains from Central Asia (Uzbekistan, Tajikistan), East Asia (China), and the Middle East, enabling comparative assessment of the Kazakhstani isolates.

**Figure 2:**
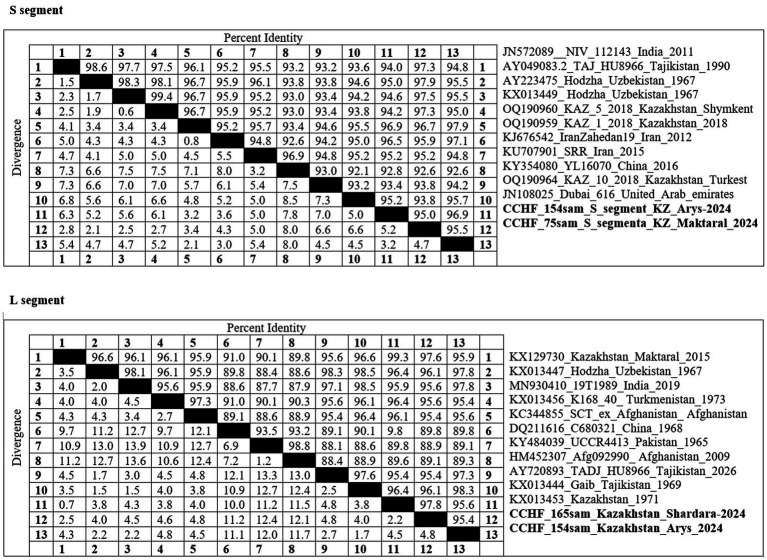
A pairwise identity matrix was constructed for the S (top) and L (bottom) segments of Crimean-Congo hemorrhagic fever virus (CCHFV) based on sequence comparisons with reference strains retrieved from GenBank. The strains highlighted in bold indicate isolates obtained in the present study.

Pairwise nucleotide identity values are presented in Figure 2 for both the S and L segments. The sequences from the present study demonstrated 92.1–98.4% identity with regional strains from Uzbekistan, Tajikistan, and China, confirming their affiliation with the Asia-1 and Asia-2 genotypes. The high similarity with strains circulating in neighboring countries supports the hypothesis of transboundary virus exchange via livestock or tick migration.

To investigate the evolutionary relationships of the CCHFV strains detected in this study, phylogenetic trees were constructed based on partial nucleotide sequences of the S and L genome segments. Representative PCR-positive samples from the Turkestan region—CCHF_15407_KZ_Maktaaral_2024 (S segment) and CCHF_ Maktaaral_2024 (L segment)—were sequenced and analyzed alongside reference strains retrieved from GenBank.

The phylogenetic analysis of the S segment is shown in Supplementary Figure 1 demonstrated that the Kazakhstani isolate clustered within the Asia-1 genotype, showing close genetic relatedness to strains from Uzbekistan (AY277437, Hoftha, 1967) and India (JN572089, 2011). The high sequence identity (96.1–96.7%) and short evolutionary distance support the hypothesis of a shared regional lineage with persistent circulation in Central Asia.

In contrast, the tree based on the L segment (see Supplementary Figure 2) showed that the Kazakhstani isolate from CCHF_154sam S segment Kazakhstan_Arys_2024 grouped with strains from Asia-2 genotype, including reference sequences from Turkmenistan (KX013463), Afghanistan (MH128201), and Uzbekistan (AY347889). This clade suggests a distinct but geographically proximate lineage that likely reflects long-term endemicity and cross-border virus maintenance.

Together, these results indicate that multiple genetic variants of CCHFV are co-circulating in southern Kazakhstan. The phylogenetic placement of the isolates within Asia-1 and Asia-2 genotypes, and their clustering with strains from neighboring countries, provide compelling evidence of transboundary virus transmission, potentially facilitated by livestock trade and the movement of infected tick vectors.

The phylogenetic relationships were inferred using the Tamura-Nei substitution model and a heuristic search with nearest-neighbor interchange (NNI) in MEGA version 12. The isolate obtained in this study (CCHF_165_KZ_Shardara_2024) is highlighted in bold and clusters within the Asia-1/2 genotype clade. Bootstrap values (>70%) are indicated at major nodes. Reference sequences representing recognized genotypes (Europe 1–2, Asia 1–3, Africa 2–3) were included to illustrate the genetic positioning of the Kazakhstani isolate within the global diversity of CCHFV.

The tree was constructed using the Tamura-Nei substitution model and the maximum likelihood method in MEGA version 12, with bootstrap support values (>70%) indicated at key nodes. The isolates obtained in this study (CCHF_154sam_KZ_Arys_2024 and CCHF_75sam_KZ_Maktaaral_2024) are shown in bold and cluster within the Asia-2 genotype. Reference sequences from GenBank representing major global genotypes (Asia 1–2, Africa 1–3, Europe 1–3) were included to contextualize the phylogenetic placement of the Kazakhstani strains within the broader genetic diversity of CCHFV.

## 4 Discussion

This study provides one of the first large-scale assessments of arboviral infections in ixodid ticks collected from livestock in southern Kazakhstan. The inclusion of 3,281 ticks collected across three ecologically distinct regions of southern Kazakhstan enhances the statistical power and geographic representativeness of our findings. Our findings emphasize the epidemiological significance of *Dermacentor* and *Hyalomma* ticks as potential vectors of CCHFV in this region. Notably, the exclusive detection of CCHFV RNA—while BTV, SBV, and LSDV were not identified—further underscores the prominent role of this virus in the regional arboviral landscape.

The absence of BTV, SBV, and LSDV in the examined samples may reflect several factors. First, these viruses are known to have limited vector competence among hard ticks; instead, they are predominantly transmitted by Culicoides midges (BTV and SBV) or hematophagous flies and mosquitoes (LSDV).

Second, the study period and environmental conditions may not have coincided with active transmission seasons for these viruses (36, 37). Third, the epidemiological situation in the region suggests that these arboviruses are either not endemic or sporadic in circulation in Kazakhstan, as no major outbreaks were reported in 2024. These findings are consistent with earlier regional reports, which also failed to detect BTV or SBV RNA in tick samples from similar ecological zones.

Comparative data from other endemic regions strengthen the interpretation of our results. For example, studies conducted in Turkey and Iran have demonstrated similar patterns of CCHFV dominance among tick-borne viruses, with *H. marginatum* and *H. anatolicum* as primary vectors. In Pakistan, *H. anatolicum* has also been identified as the major vector responsible for transmitting CCHFV to both livestock and humans, particularly in arid and semi-arid zones (38, 39). The dominance of hard ticks in the sample may have biased results toward detection of tick-borne viruses only. Tick sampling was conducted from April to early June 2024, which may not fully overlap with peak transmission seasons for BTV and SBV. These findings align with our detection of CCHFV in *H. anatolicum* and *H. scupense* from southern Kazakhstan, highlighting the importance of regional tick species as virus reservoirs.

The predominance of female *D. pictus, H. anatolicum,* and *D. marginatus* among CCHFV-positive pools aligns with previous reports on sex-specific differences in infection rates, likely reflecting extended host attachment and greater blood meal volume in female ticks, which may enhance their vector competence (40, 41). These observations support the hypothesis that sex- and species-specific biological differences may influence virus maintenance, with female *Dermacentor* and *Hyalomma* ticks acting as potential reservoirs. Importantly, to reduce potential bias, tick sampling across all regions followed a standardized collection protocol involving comparable livestock species and herd sizes, with district-level stratification applied in Turkestan to increase spatial resolution. However, it is important to note that our findings do not confirm vectorial competence, which requires controlled transmission studies. The geographic heterogeneity of infection, with CCHFV detected only in ticks from the Turkestan region, is particularly noteworthy. This region is known to harbor natural foci of CCHFV and has historically reported the highest incidence of human cases in Kazakhstan. Environmental conditions, livestock density, and the presence of competent tick vectors likely contribute to the region’s role as an arboviral hotspot (42). However, RNA detection alone does not confirm vector competence and should be interpreted with caution. Additionally, the calculated positivity rates reflect pooled sample testing, and not individual tick infection rates, which may lead to an overestimation of true prevalence. These findings are consistent with earlier ecological modeling studies, which identified southern Kazakhstan as a high-risk area for CCHFV circulation (43, 44).

Phylogenetic analysis of partial S and L segments revealed that the CCHFV isolates from Turkestan belonged to the Asia-1 and Asia-2 genotypes, clustering closely with historical strains from Uzbekistan, Turkmenistan, and China. These results provide compelling molecular evidence for transboundary virus exchange across Central Asia. Livestock movement, shared grazing pastures, and informal trade likely facilitate cross-border viral transmission, as has been proposed in previous cross-sectional studies conducted along the Kazakh–Uzbek border.

The genetic divergence observed between the S and L segment-based phylogenies suggests the potential for segment reassortment or co-circulation of multiple genotypes within the same geographic focus. While no evidence of recombination was observed in the current study, further whole-genome sequencing and longitudinal sampling would be needed to clarify the evolutionary dynamics of CCHFV in Kazakhstan.

The infection status of host animals was not assessed, which limits interpretation of virus circulation at the host–vector interface. Importantly, the present work fills a critical gap in regional epidemiological surveillance and provides a baseline for future vector monitoring programs. In the absence of systematic screening of ticks for arboviruses, the actual extent of virus circulation in livestock-associated ecosystems remains poorly understood. Our approach—combining field collection, molecular detection, and phylogenetic resolution—offers a scalable model for regional risk assessment.

While the current study provides a robust snapshot of CCHFV circulation in livestock-associated ticks, longitudinal data will be essential to understand temporal trends, emergence of novel genotypes, and their public health relevance. Integrating entomological surveillance with climatic and land-use data may also improve predictive modeling of arboviral risks in the region.

Future studies should aim to expand tick sampling to additional ecological zones and host species, including wildlife reservoirs; integrate seroepidemiological data from livestock and humans to map exposure risk; and assess viral load and infection dynamics within vector tissues to better define vector competence at the species level.

Overall, this study demonstrates the value of molecular surveillance for early detection of zoonotic arboviruses and highlights the importance of cross-border collaboration for the containment of vector-borne threats in Central Asia.

## 5 Conclusion

This study provides the first integrated molecular surveillance of arboviral pathogens in ixodid ticks collected from livestock in southern Kazakhstan. Among the four targeted arboviruses—CCHFV, BTV, SBV, and LSDV-only CCHFV RNA was detected, with the highest infection rates observed in female *D. pictus, H. anatolicum*, and *D. marginatus* ticks from the Turkestan region. These findings highlight the species-specific and regional heterogeneity of arboviral circulation and identify key tick vectors potentially involved in maintaining CCHFV transmission cycles.

Phylogenetic analysis confirmed the affiliation of Kazakhstani isolates with Asia-1 and Asia-2 genotypes and revealed close genetic relatedness to strains circulating in neighboring countries. This supports the hypothesis of transboundary virus exchange and underscores the need for coordinated regional surveillance. These findings underscore the need for enhanced regional surveillance of arboviral pathogens in ticks and livestock. We recommend the establishment of integrated surveillance programs combining molecular screening of tick vectors and serological monitoring of livestock, particularly in ecologically high-risk areas such as the Turkestan region. Additionally, targeted vector control strategies and intersectoral collaboration should be considered as part of a One Health approach. Overall, the results of this study provide valuable baseline data for future epidemiological monitoring and contribute to a better understanding of the ecology, distribution, and genetic structure of CCHFV in Central Asia.

## Data Availability

The data presented in the study are deposited in the GenBank repository, accession numbers PV646616–PV646619.
