# Supplementary material for: Genetic evidence of regional circulation of Crimean-Congo hemorrhagic fever virus in ixodid ticks from southern Kazakhstan
Source: Front Vet Sci. 2025 Oct 3;12:1623822. doi: 10.3389/fvets.2025.1623822 (PMC12532773; doi:10.3389/fvets.2025.1623822)
Supplement: Supplementary file 1 [file Data_Sheet_1.DOCX]

***Supplementary Material***

***
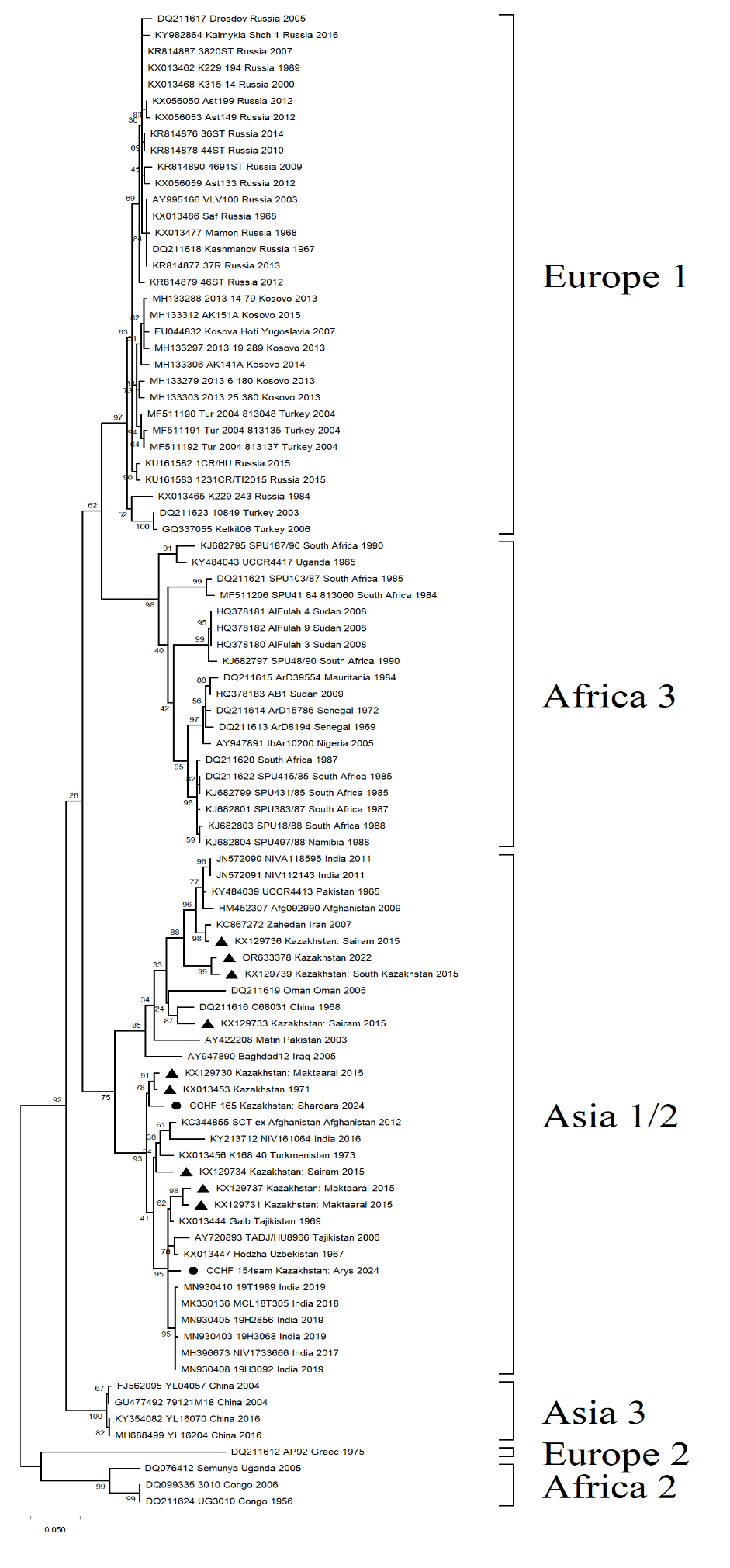
***

Supplementary Figure 1. Maximum likelihood phylogenetic tree based on partial S segment sequences of CCHFV. The isolate obtained in this study (CCHF_165 KZ_Shardara_2024) is included in the tree. Legend: ■ Asia-1, ● Asia-2, ▲ Africa-3, ◆ Africa-1, ▼ Europe 1/2. Bootstrap values ≥70% are shown at key nodes.


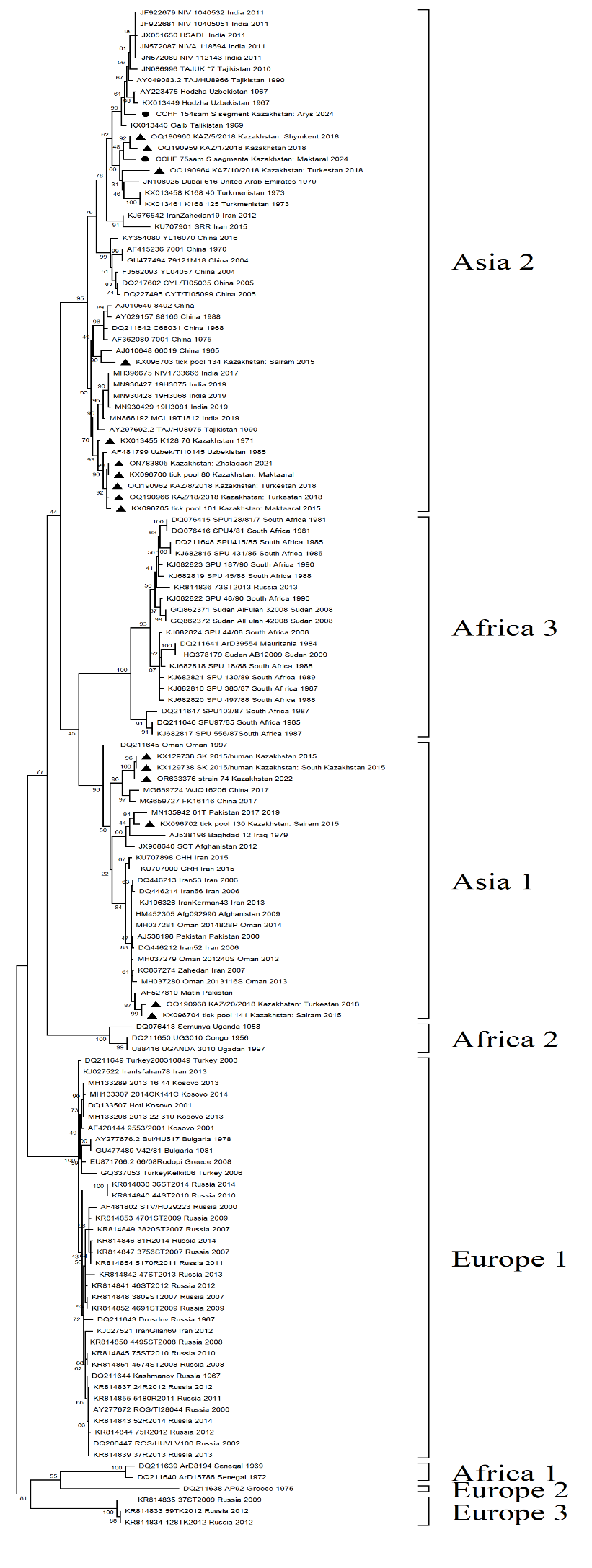


Supplementary Figure 2. Maximum likelihood phylogenetic tree based on partial L segment sequences of CCHFV. The study isolates (CCHF_154sam KZ_Arys_2024 and CCHF_75sam KZ_Maktaral_2024) are included in the tree. Legend: ■ Asia-1, ● Asia-2, ▲ Africa-3, ◆ Africa-1, ▼ Europe 1/2. The isolates cluster within the Asia-2 genotype.
